# Retraction Note: Bone Marrow Mesenchymal Stem Cell-Derived Exosomal MicroRNA-133a Restrains Myocardial Fibrosis and Epithelial–Mesenchymal Transition in Viral Myocarditis Rats Through Suppressing MAML1

**DOI:** 10.1186/s11671-022-03764-7

**Published:** 2022-12-09

**Authors:** Qiming Li, Yunpeng Jin, Xiaoqi Ye, Wei Wang, Gang Deng, Xiaojian Zhang

**Affiliations:** 1 The Department of Cardiology, The Fourth Affiliated Hospital of Zhejiang University School of Medicine, N1 Shangcheng Road, Yiwu, 322000 Zhejiang China; 2 Nursing Department, The Fourth Affiliated Hospital of Zhejiang University School of Medicine, Yiwu, 322000 Zhejiang China; 3 The Ningbo Central Blood Station, Ningbo, 315040 Zhejiang China

**Retraction Note to: Nanoscale Res Lett (2021) 16:111**

https://doi.org/10.1186/s11671-021-03559-2

The Editors in Chief have retracted this article. After publication, concerns were raised regarding the unlikely appearance of the data presented in the flow cytometry histograms in Fig. 1D. The authors did not respond to the request to comment and provide raw data or evidence of the ethics approval. The editors, therefore, have lost confidence in the integrity of the article's findings. The editors were not able to obtain a current email address for Author Gang Deng. Authors have not responded to any correspondence from the editor or publisher about this retraction.

